# Draft genome sequence of *Intrasporangium* sp. DVR, an actinobacterium of the family *Intrasporangiaceae* isolated from soil in Japan

**DOI:** 10.1128/mra.00413-24

**Published:** 2024-06-11

**Authors:** Seigo Amachi, Nobuyoshi Nakajima, Shigeki Yamamura

**Affiliations:** 1 Graduate School of Horticulture, Chiba University, Matsudo, Japan; 2 Biodiversity Division, National Institute for Environmental Studies, Tsukuba, Japan; 3 Regional Environment Conservation Division, National Institute for Environmental Studies, Tsukuba, Japan; Department of Biology, Queens College, Queens, New York, USA

**Keywords:** intrasporangium, draft genome

## Abstract

*Intrasporangium* sp. strain DVR is an actinobacterium of the family *Intrasporangiaceae* isolated from soil in Japan. Here we report the draft genome sequence of strain DVR.

## ANNOUNCEMENT

The genus *Intrasporangium* belongs to the phylum *Actinomycetota* and comprises five species, that is, *I. calvum*, *I. chromatireducens*, *I. flavum*, *I. mesophilum*, and *I. oryzae* (1). They are known as Gram-positive aerobic bacteria but sometimes show capacities for nitrate reduction under anaerobic growth conditions (2, 3). Thus, this taxonomic group may catalyze anaerobic metabolisms in nature. Members of this genus have been isolated from various types of soil, but little is known about the ecophysiological functions of this group of bacteria. Genome information is important for the development of a thorough understanding of the metabolic potential of this taxonomic group. Here, we report a draft genome sequence of an *Intrasporangium* bacterial strain DVR isolated from soil in Japan.

Strain DVR was isolated from the soil of a public park, Ichikawa City, Chiba, Japan (35°40′N, 139°54′E) using extinction dilution and a single-colony isolation technique. Strain DVR was grown anaerobically in a minimum medium (4) containing lactate (10 mM) as the sole carbon source. The genomic DNA was extracted using a DNeasy Blood and Tissue Kit (Qiagen, Hilden, Germany). A paired-end DNA library (insert size, 75–700 bp) was constructed using a TruSeq DNA PCR-free LT Sample Prep Kit (Illumina, CA, USA). Sequencing of the library with an Illumina Miseq System (Illumina) generated 3,180,354 reads with an average length of 214.19 bp. Low-quality sequences (<Q20) and adopters were trimmed using the Trim Reads Tool of CLC Genomics Workbench v. 23.0.4 (CLC-GWB; Qiagen). The resulting 3,015,354 reads (587,345,747 bp in total) were assembled using the *De Novo* Assembly Tool of CLC-GWB with the following parameters: automatic bubble size: yes, minimum contig length: 500, word size: 40, perform scaffolding: yes, auto-detect paired distances: yes, mismatch cost: 2, insertion cost: 3, deletion cost: 3, length fraction: 0.5, and similarity fraction: 0.8. The completeness of the assembly was evaluated using BUSCO v4.0.2 software (5) with data set micrococcales_odb 10 as reference data. A total of 537 BUSCO groups were identified, of which 534 (99.5%) were complete BUSCOs, one was a fragmented BUSCO, and two were duplicated BUSCOs. The draft genome sequence (Table 1) consisted of 3,821 coding sequences, 3 ribosomal RNAs, and 52 transfer RNAs as predicted by DDBJ Fast Annotation and Submission Tool (DFAST) v. 1.2.18 (6). The BLASTN analysis of the full-length 16S rRNA gene revealed that the closest relative of strain DVR is *I. calvum* DSM 43043^T^ (NR_074507) with a nucleotide sequence identity of 99.1%, suggesting that strain DVR is a member of the genus *Intrasporangium*. Default parameters were used for all software unless otherwise specified.

**TABLE 1:** General genomic features of *Intrasporangium* sp. DVR

Size (bp)	Coverage (×)	GC (%)	Contigs	*N*_50_ (bp)	CDSs	Protein-coding genes	Genome accession no.	SRA accession no.
4,135,220	142	70.8	61	303,653	3,821	3,766	BAABZW000000000.1	DRR536540

## Data Availability

The draft genome sequence of *Intrasporangium* sp. DVR has been deposited in NCBI/ENA/DDBJ under BioProject and BioSample accession numbers PRJDB17667 and SAMD00755701, respectively. GenBank and SRA accession numbers are available in Table 1.
